# Sex Hygiene for the Male, and What to Say to the Boy

**Published:** 1912-04

**Authors:** 


Sex Hygiene for the Male, and What to Say to the Boy.—By G. Frank Lydston, M. D., Professor of the Surgical Diseases of the Genito-Urinary Organs and Syphilology, Medical Department, State University of Illinois; Member of the American Medical Association; Member of the American Urological Association; Member of the Society of Authors of London, England; Author of "Diseases of Society," "The Blood of the Fathers," "Over the Howkale," etc. Delegate from the United States Government to the Congress for the Prevention of Infectious Diseases, Brussels, Belgium, etc., etc. Illustrated with twenty-four engravings. Chicago: The Riverton Press. 1912. Price not stated.

Here is another one of Dr. Lydston's characteristic works, fully up to the standard of its predecessors. His writings along this line —more especially his "Diseases of Society"—have met with enthusiastic reception by the press and the profession of which he is a brilliant and popular representative. It is most timely, inasmuch as the subject of sex hygiene—an important factor in the propaganda for social reform and prophylaxis of crime and other forms of race degeneracy—is now foremost. It "holds the boards" and is being discussed by all exceptional men and women and by the more enlightened and liberal of the medical and popular magazines. They see and appreciate the necessity of popular education on this heretofore tabooed subject if ever we are to break down the barrier of false modesty, prejudice and ignorance, and make people take their fingers from their faces and look at and talk of the dangers of the venereal plague, so destructive, not only to the individual, but to the family and the race. Promiscuous indulgence of the basic impulse is the greatest evil that besets society, unless I except the infernal saloon, and the young should be taught to avoid it. And who should teach them but the parents? True, it should be taught in all schools. Boys and girls, at puberty or before, feel the promptings of the procreative instinct, at a time when the moral sense, hence the inhibitive power, is not developed, and they too often resort to natural and unnatural methods of gratification, with direful results. It is arrant nonsense to say that a father should not talk to his son about that which most nearly concerns, not only his health and happiness, but his mission in life, parenthood and family. It is a mockish sentimentality with which I have no patience. The subject is not "indelicate"—it is of first importance. Dr. Lydston long ago realized this important fact, and has done much towards reform. His present book is timely and important; and I wish every boy in this broad land could read it. The TEXAS MEDICAL JOURNAL commends it in the highest terms, and extends its thanks and congratulations to the versatile author.

I quote the doctor's dedication: "To the parents and teachers who sincerely wish to fight for our boys and who wish to learn what to say to them, and to the physician who wishes to lighten his labors by educating the public in matters pertaining to sex."

The boys of today will be the men of tomorrow, who must carry on the work of the world for the advancement of civilization. F. E. D.

---

In intestinal obstruction, it is not the operation that is to be feared, but the delay in operation.—*American Journal of Surgery.*

---

There is no convincing argument in favor of amputating *normal* omentum found in a hernia. There are sound arguments against it.—*American Journal of Surgery.*

